# The IL-1 inhibitor Canakinumab for Familial Mediterranean Fever: the Greek experience in 12 patients

**DOI:** 10.1186/1546-0096-13-S1-P72

**Published:** 2015-09-28

**Authors:** K Laskari, P Boura, GN Dalekos, A Garyfallos, D Karokis, D Pikazis, L Settas, G Skarantavos, E Tsitsami, PP Sfikakis

**Affiliations:** 1 Athens University Medical School, Rheumatology Unit, 1st Dept. of Propaedeutic Internal Medicine, Athens, Greece; 2 Aristotle University Medical School, Clinical Immunology Unit, 2nd Dept. of Internal Medicine, Thessaloniki, Greece; 3 Thessaly University Medical School, Dept. of Medicine and Research Laboratory of Internal Medicine, Larissa, Greece; 4 Aristotle University Medical School, 4th Dept. of Internal Medicine, Thessaloniki, Greece; 5 Private rheumatologist, Patras, Greece; 6 Athens University Medical School, Department of Pathophysiology, Athens, Greece; 7 Aristotle University Medical School, First Dept. of Internal Medicine, Rheumatology Section, Thessaloniki, Greece; 8 Athens University Medical School, Bone Metabolic Unit, 1st Dept. of Orthopedics, Athens, Greece; 9 Athens University Medical School, 1st Dept. of Pediatrics, Athens, Greece

## Background

IL-1 is a major mediator of the inflammatory cascade in Familial Mediterranean Fever (FMF) and an established therapeutic target [1].

## Objective

To retrospectively assess the efficacy and safety of the IL-1 inhibitor Canakinumab in adult and adolescent FMF patients, including cases resistant to the IL-1 receptor antagonist anakinra.

## Methods

Twelve patients (7 men) with genetically confirmed FMF, fulfilling the Tel Hashomer criteria, aged 32.5 years (median, range 13-70), with median disease duration of 168 months and active disease refractory to colchicine (n=8) and/or anakinra (n=4), received Canakinumab 150mg subcutaneously every 4 (n=7) or 6 (n=3) or 8 weeks (n=2) for a median of 12 months (range 4-46). Canakinumab was given as monotherapy in 9; 3 patients received concomitant treatment with colchicine and/or corticosteroids. Clinical and laboratory parameters during follow-up were recorded.

## Results

Seven out of 12 patients (58%) achieved complete clinical remission within a median time of one month. Normalization of all laboratory parameters associated with inflammation occurred in 70% of patients within a median time of 2 months and in all, but one, of these patients complete clinical remission was also reached. Response was maintained until the last visit in all patients with clinical and/or serological complete remission. The remaining patients achieved partial responses, with persisting, albeit milder, arthralgias and abdominal pain, and lower, but abnormal CRP levels. Overall, the concomitant corticosteroid dose was significantly reduced during follow up. The recently proposed FMF50 score for assessing outcome in FMF (2) was achieved by 67% and 88% of patients at one month and 12 months, respectively. Canakinumab was well tolerated; one patient experienced an urinary tract infection, which resolved with antibiotics.

## Conclusion

The rapid and sustained response to Canakinumab in the majority of our patients, together with the favorable safety profile, encourages its further use in FMF.
